# O19 A case of pyogenic arthritis, pyoderma gangrenosum and acne syndrome

**DOI:** 10.1093/rap/rkab067.018

**Published:** 2021-10-19

**Authors:** Diarmuid McLaughlin, Dearbhla McKenna, Cathy Campbell, Madeline Rooney

**Affiliations:** Department of Paediatric Rheumatology, Musgrave Park Hospital, Belfast, United Kingdom

## Abstract

**Case report - Introduction:**

Considering an auto-inflammatory disorder is important where recurrent fevers are a presenting feature. Performing a genetic panel is essential in these cases; however, it can be negative even in the presence of an underlying auto-inflammatory disorder. Our case highlights the importance of this with a reminder that a negative gene panel does not completely exclude an underlying auto-inflammatory disorder in the presence of suggestive history and clinical examination findings.

**Case report - Case description:**

A 5-year-old girl presented with recurrent episodes of fever (>38 °C), abdominal pain and joint pains (back, wrist, hip, knee & ankle). Past medical history included episodes of self-resolving neutropenia. Initial clinical examination revealed mouth ulcers, a significant number of dental caries, a petechial rash and genital erythema. There was no active joint disease. A pustular like itchy rash developed on/off over time affecting the buttocks, genital area, knees and elbows. Episodes increased in frequency becoming once per month, lasting for 5 days on average, resulting in a significant amount of time off school.

Initial differential diagnosis included periodic fever and cyclical neutropenia. White blood cells ranged from 4.3 to 20.1 (10^9/l), Neutrophils ranged from 1.1 to 11.4 (10^9/l), CRP 0.7 to 149.5 (mg/L), ESR 2 to 15 (mm/hr). Serum Amyloid A ranged from 8.0 to 370 (mg/L). Immunology and haematology consults were sought with cyclical neutropenia deemed unlikely. A primary immunodeficiency screen was negative. Initial genetic analysis for periodic fever was negative; however, this was later reanalysed and revealed a pathogenic mutation in the PSTPIP1 gene and a mutation in the TMEM173 gene. A mutation in the PSTPIP1 gene, on chromosome 15q24 is associated with Pyogenic Arthritis, Pyoderma gangrenosum and Acne (PAPA) Syndrome.

A trial of colchicine was commenced, with eventual titration of the dose, achieving a good response. Occasional episodes now occur 1—2-times monthly with a reduced temperature rise, minimal arthralgia and are of a shorter duration. Dental extraction was also performed due to 9 periodontal abscesses. Skin care advice included use of emollient-barrier creams, antihistamines and oral antibiotics when indicated.

**Case report - Discussion:**

PAPA – Pyogenic Arthritis, Pyoderma Gangrenosum and Acne are a triad of symptoms that together combine to form this rare auto-inflammatory disorder. It is an autosomal dominant condition. It is the result of a mutation of the (PSTPIP1/CD2BP1) gene located on chromosome 15. Studies of families affected have revealed a variable penetrance, including some asymptomatic carriers. Clinicians should be mindful when ascertaining what conditions and symptoms other family members have, as PAPA could be the underlying causative condition. The recurrent aseptic inflammation of the joints normally manifests within the first two decades of life. The skin symptoms manifest within the third decade. All three symptoms rarely appear instantaneously. The diagnosis is clinical with confirmation by genetic testing. Reported treatments includes the use of corticosteroids, arthrocentesis – for relieving pain, and biologics – namely anti IL1 and anti TNF therapy.

We felt this case was interesting as the patient described demonstrated a good response to colchicine and highlighted the difficulties that can present in making the diagnosis. With a confirmed diagnosis the clinician can then appropriately follow up the patient, giving them and potential family members the best care available.

**Case report - Key learning points:**

Our case highlights the importance of multi-specialty input particularly when faced with a rare or complex case in order to exclude other potential causes. Although genetic testing has significantly improved in helping with the diagnosis of auto-inflammatory disorders, it must be highlighted the importance of good clinical assessment and interpretation of more standard investigations. This is particularly relevant where genetic panelling is returned as negative and concerns persist of an underlying auto-inflammatory disorder. We hope to raise awareness of the varied presentation of PAPA syndrome particularly in the younger age groups where skin presentations may not be a predominant presenting feature. It is also important to emphasise that the main features of PAPA syndrome rarely present simultaneously. Current clear treatment pathways do not exist due to the rarity of this condition within the paediatric population – a number of agents are suggested in the literature with colchicine achieving good control in our patient described.

